# Smoking prevalence and associated risk factors among healthcare professionals in Nicosia general hospital, Cyprus: a cross-sectional study

**DOI:** 10.1186/s12971-016-0079-6

**Published:** 2016-04-07

**Authors:** Stavri Zinonos, Theodora Zachariadou, Savvas Zannetos, Andrie G. Panayiotou, Andreas Georgiou

**Affiliations:** Health Center of University of Cyprus, Cyprus International Institute for Environmental and Public Health in association with the Harvard T.H. Chan School of Public Health, Cyprus University of Technology, 1, Panepistimiou Avenue, 2109 Aglantzia, Nicosia Cyprus; Open University of Cyprus, 33 Giannou Kranidioti Avenue, 2220 Latsia, Nicosia Cyprus; Cyprus International Institute for Environmental and Public Health in association with the Harvard T.H. Chan School of Public Health, Cyprus University of Technology, 95 Eirinis Street, 3041 Limassol, Cyprus; Respiratory Department Clinic, Nicosia General Hospital, 215 Nicosia – Limassol Old Road, 2029 Strovolos, Nicosia Cyprus

**Keywords:** Smoking prevalence, Healthcare professionals, Smoking attitudes

## Abstract

**Background:**

In recent years, a significant progress has been achieved globally in reduction of smoking among physicians and nurses, however, in some countries the smoking prevalence of health professionals is maintained at very high levels, without significant difference from the general population. This study aims to investigate the prevalence of smoking among physicians and nurses working at Nicosia General Hospital, as well as their knowledge and attitudes towards smoking cessation strategies.

**Methods:**

This is a cross-sectional questionnaire-based study. The study consisted of 119 doctors and 392 nurses currently working at Nicosia General Hospital in Cyprus. Study participants were recruited from all hospital wards between May and June 2008. Both physicians and nurses were asked to answer an anonymous questionnaire, which included questions regarding their smoking habits, knowledge and attitudes about smoking and smoking cessation strategies.

**Results:**

Overall smoking prevalence among healthcare professionals was 28.2 % (28.6 % among physicians and 28.1 % among nurses). Multivariate analysis revealed that being male, younger than 34 years old, unmarried and with a family history of smoking were associated with increased likelihood of being a current smoker. An impressive 72 % of current smokers reported that they wished to quit smoking, however, only 5.6 % of physicians and 6.9 % of nurses, reported ever using any smoking cessation aids. Never- smokers counseled their patients to quit smoking more often (96.4 %) compared to former (84.6 %) and current smokers (72.7 %), (*p* < 0.001). In addition, those who felt more confident about their knowledge regarding smoking cessation, reported counseling their patients to quit smoking more often compared to those who did not (92 % vs 60 %, *p* < 0.001).

**Conclusions:**

Smoking prevalence among physicians and nurses working at Nicosia General Hospital was similar to that of the general Cypriot population. Further training of healthcare professionals towards smoking cessation strategies is needed in order to improve their knowledge and consequently their efforts on counseling and support to their patients who wish to quit smoking.

## Background

Tobacco use has been generally acknowledged as one of the biggest public health threats ever faced, with more than one billion smokers worldwide [1]. According to the World Health Organization (WHO), smoking kills nearly 6 million people annually, an average of one person every six seconds, and accounts for 6 and 12 % of all female and male deaths respectively [1, 2]. In addition, in 2014, World Health Organization after the revision of relevant data regarding smoking, reports that tobacco use is the direct cause of more than five million deaths, while more than 600,000 deaths are attributable to second-hand smoking [1]. If current trends continue and drastic measures are not taken to control tobacco use, annual tobacco deaths are expected to rise to 10 million worldwide by 2030 [2]. The biggest impact of smoking is through non-communicable diseases, although it may also increase the risk of communicable diseases such as tuberculosis and lower tract respiratory infections [3]. It is responsible for 87 % of all lung cancer deaths [4], 42 % of chronic respiratory diseases and nearly 10 % of cardiovascular diseases [5].

Healthcare professionals should be active in smoking prevention, as part of their daily practice [6]. They should discuss with their patients the harmful effects of smoking and available smoking cessation interventions and support them through any smoking cessation process [7–11]. There is evidence that when physicians and nurses offer specific assistance and appropriate support, a large percentage of their patients who smoke will try to quit, even those with low motivation to quit [12]. Furthermore, the smoking status of healthcare professionals appears to be a critically important determinant of their ability to assist their patients to control tobacco use. Studies suggest that as smoking rates among physicians decline, a similar reduction will be observed in the general population as well [13]. However, while in most developed countries the rate of physicians and nurses who smoke has declined significantly over the last 30 years [14], in other countries it has been maintained at very high levels and without a significant difference to that of the general population [14].

In Cyprus, smoking prevalence among the general population is 26.5 % [9] and so far, no studies have been performed regarding the prevalence of smoking among physicians and nurses. This study aims to estimate smoking prevalence among physicians and nurses working at Nicosia General Hospital (NGH), the largest hospital in Cyprus and to investigate their knowledge and attitudes towards smoking and smoking cessation strategies, as well as possible interactions with their own smoking status.

## Methods

### Study population

This cross-sectional study included 119 doctors and 392 nurses working at NGH, in Cyprus. Study participants were recruited from all hospital wards between May and June 2008. A self - completed anonymous questionnaire was administered to all participants after signing a consent form. The NGH is the largest hospital of the island providing tertiary care and is the referral hospital of all other hospitals and clinics of the island. Therefore, the study sample could be considered representative of public sector physicians and nurses.

### Sample size calculations and recruitment

All physicians working at NGH were asked to participate in the survey (*n* = 175) [15]. Twenty-nine of them were absent at the time of the survey. Further, 27 refused to participate, thus the response rate for physicians was 81.5 %.

For nurses, a sample size calculation was performed as follows. For a hypothesized smoking prevalence of 26 % (population average), with a Confidence Level of 95 %, a margin of error of ± 5 % and no Finite Population Correction application, a sample of 296 nurses was needed. A conservative 40 % non-response rate, due to no prior experience on similar studies, was added, giving a total sample size of 493 nurses. From the total of 852 [16] nurses working at NGH, 493 were randomly selected among all wards of the hospital using a hospital payroll list with a unique consecutive number attached to each name and the random sample command in SPSS was used to select the appropriate random sample. At the time, 23 nurses were absent and did not participate. Out of the 493 randomly selected nurses, 392 agreed to participate giving a response rate of 79.5 %.

### Questionnaires

Both physicians and nurses were sought out on a one-to-one basis by the study researchers and asked to answer an anonymous questionnaire, which was taken from a Greek study by Sotiropoulos et al. [7]. The questionnaire included questions regarding smoking history, attitudes about smoking and smoking cessation as well as the demographic characteristics of the participants.

Regarding smoking status, respondents were classified as current smokers, former smokers and never-smokers. Participants were asked to state how often they counsel patients to stop smoking (rarely/never/sometimes/often), about their attitude as to whether healthcare professionals should undergo specific training to help their patients who wish to quit smoking (Yes/No) and about their attitude as to whether smoking should be prohibited in hospitals and in public places (Yes/No).

Nicotine Dependence was evaluated with the use of the Fagerstrom Nicotine Tolerance Questionnaire (FTQ) [17]. Smokers were divided into three categories: low nicotine dependence, medium nicotine dependence and high nicotine dependence following the recommended procedure and cut off points from the creators of the questionnaire.

### Definitions

As current smokers were defined those who reported smoking at least 100 cigarettes in their lifetime and who, at the time of survey, smoked either every day or some days. Αs former smokers were defined those who reported smoking at least 100 cigarettes in their lifetime and who, at the time of the survey, did not smoke at all. Respondents who reported never having smoked 100 cigarettes, were defined as never smokers [18]. The family history of smoking was determined with the question “Does anyone else smoke in the family?”

On the basis of the FTQ, smokers were divided into low nicotine dependent category (0 ≤ FTQ score ≤3), medium nicotine dependent (4 ≤ FTQ score ≤6) and high nicotine dependent (7 ≤ FTQ score ≤10) [17]. Pack-years of smoking were defined by multiplying the number of cigarettes smoked per day by the number of years the person has smoked and then divided by 20 (cigarettes of one pack).

### Statistical analysis

Continuous variables were expressed as medians (after checking their distribution), while nominal variables were expressed as frequencies and percentages. Chi-square test or Fisher’s exact test, were used to examine the association between variables. Moreover, Mann–Whitney test was used to examine the statistical relationship among non-normally distributed data. A multiple forward stepwise logistic regression analysis was used to determine independent predictors of the likelihood of being current smoker vs. never - smokers. The covariates examined were gender, age, marital status, having children and family history of smoking. Indicator variables were created for all categorical variables and odds ratios with 95 % confidence intervals (CI) were presented. Level of statistical significance was defined at α = 0.05 therefore, p value < 0.05 was required for a covariate to be in the model. No interaction effects between covariates were statistical significant, thus not included in the model. In addition, for the logistic regression, due to small sample size of the variable marital status, the categories unmarried, divorced and widowed were combines into one group.

All statistical analysis was performed using the IBM SPSS Statistics Software, version 20.0.

## Results

### Characteristics of the study population

From the total 511 participants, 119 were physicians (23.3 %) and 392 were nurses (76.7 %). The participants aged 25–34 years old represented 41.7 % of the study population. Sixty three percent of physicians and 68.9 % of nurses were married. Detailed characteristics of the study population are presented in Table 1.

**Table 1:** Characteristics of the study population (*n* = 511)

	All		Physicians		Nurses		*p* value
	N	%	N	%	N	%	
Sex							
Male	139	27.2	71	59.7	68	17.3	<0.001
Female	372	72.8	48	40.3	324	82.7	
Age (years)							
25–34	213	41.7	48	40.3	165	42.1	0.925
35–44	111	21.7	28	23.5	83	21.2	
45–54	135	26.4	30	25.2	105	26.8	
55–64	52	10.2	13	10.9	39	9.9	
Does anyone else smoke in the family?							
Yes	99	19.4	30	25.2	69	17.6	0.066
No	412	80.6	89	74.8	323	82.4	
Smoking Status							
Current	144	28.2	34	28.6	110	28.1	0.002
Former	42	8.2	19	16.0	23	5.9	
Never	325	63.6	66	55.5	259	66.1	
FTQ-Level of Dependence							
Low nicotine dependent	87	61.3	20	58.8	67	62.0	0.385
Medium nicotine dependent	39	27.5	8	23.5	31	28.7	
High nicotine dependent	16	11.3	6	17.6	10	9.3	
Pack- years of smoking	Median(IQR)		Median(IQR)		Median(IQR)		*p* value^*^
Current smokers	7.5 (10.50)		8.5 (10.25)		7.50 (10.50)		0.857
Former smokers	5.0 (8.50)		5.0 (18.65)		1.0 (5.50)		0.061

^*^Mann Whitney test was used

### Smoking prevalence and factors associated with smoking status

The overall prevalence of smoking among healthcare professionals working at NGH was 28.2 % and it was similar among physicians and nurses (28.6 and 28.1 % respectively, *p* = 0.874). A total of 186 participants were current or former smokers (36.4 %). Among all, 16 % of doctors and 5.9 % of nurses were former smokers, whereas 55.5 % of physicians and 66.1 % of nurses had never used tobacco in their life. Moreover, 8 % of nurses and 5.9 % of physicians admitted that they smoke in front of their patients.

Factors associated univariately with smoking status were: younger age (*p* < 0.001), being male (*p* < 0.001), being single (*p* = 0.002) and having a family history of smoking (*p* < 0.001). Details are shown in Table 2.

**Table 2:** Characteristics of the study population by smoking status

	Never Smokers		Current Smokers		Former Smokers		*p* value
	N	%	N	%	N	%	
Sex							
Male	64	46	56	40.3	19	13.7	<0.001
Female	261	70.2	88	23.7	23	6.2	
Age (years)							
25–34	122	57.3	78	36.6	13	6.1	<0.001
35–44	91	82.0	15	13.5	5	4.5	
45–54	79	58.5	39	28.9	17	12.6	
55–64	33	63.5	12	23.1	7	13.5	
Marital Status							
Unmarried	73	53.3	54	39.4	10	7.3	0.002*
Married	239	69.3	77	22.3	29	8.4	
Divorced	8	42.1	9	47.4	2	10.5	
Widowed	5	50	4	40	1	10	
Does anyone else smoke in the family?							
Yes	13	13.1	78	78.8	8	8.1	<0.001
No	312	75.7	66	16	34	8.3	

*Fisher’s exact test was used

In addition, classification analysis was performed. Several algorithms (e.g., random forests, naïve Bayes) were implemented with the best (in terms of accuracy) performing algorithm to be logistic regression. Thus, in a multiple forward stepwise logistic regression model, with smoking status (current vs. never- smokers) as the dependent variable, the final fitted model included gender (*p* < 0.001), age (*p* = 0.004), marital status (*p* = 0.012) and family history of smoking (*p* < 0.001). Thus, male, unmarried, younger than 34 years old healthcare professionals with family history of smoking were the most likely to be smokers. Details are presented in Table 3.

**Table 3:** Multivariable logistic regression of smoking status against demographic characteristics

Unadjusted						Adjusted				
	B	*p* value	Odds ratio	95 % C.I. for odds ratio		B	*p* value	Odds ratio	95 % C.I. for odds ratio	
Constant	−1.087	.000	.34			−1.146	.000	0.32		
Gender										
Male	.954	.000	2.60	1.68	4.00	1.012	.000	2.75	1.59	4.75
Age (years)										
35 - 44						−1.206	.004	0.30	0.13	0.69
45 - 54						.005	.989	1.00	0.51	1.99
55–64						-.376	.447	0.69	0.26	1.81
Marital Status										
Married^a^						-.769	.012	0.46	0.25	0.85
Does anyone else smoke in the family?										
Yes						1.109	0.000	3.03	1.51	6.10

^a^compared to unmarried/widowed/divorced as a combined category

### Smoking cessation attitudes among current smokers

Out of 144 current smokers, 71.8 % reported that they would like to quit. Almost half of physicians (48.5 %) and nurses (52 %) who smoke have attempted to quit smoking in the past; however, only 5.6 % of physicians and 6.9 % of nurses reported using any smoking cessation aids. The attempt to quit smoking was successful for more than half of the physicians (62.5 %) compared to only 31.5 % of nurses (*p* = 0.025).

Univariate analysis revealed that nurses and doctors advised their patients to quit smoking at similar rates (89.6 % vs. 80.7 % respectively; *p* = 0.089). No statistically significant difference was found between physicians and nurses who rarely or never counsel their patients to quit smoking (8.4 % vs. 4.5 %; *p* = 0.089).

Details about healthcare professionals’ attitudes towards counseling patients to quit smoking, by smoking status and level of dependence are presented in Table 4. No statistical significant association was found between the level of nicotine dependence and attitudes on smoking prohibition in hospitals (*p* = 0.951) or public places (*p* = 0.287).

**Table 4:** Healthcare professionals’ attitude towards counseling patients to quit smoking by smoking status and level of dependence

	Rarely/Never		Sometimes		Often		
	N	%	N	%	N	%	*p* value
Smoking Status							
Never	3	1.2	6	2.4	244	96.4	<0.001
Current	16	11.5	22	15.8	101	72.7	
Former	3	11.5	1	3.8	22	84.6	
FTQ-Level of Dependence							
Low nicotine dependence	7	8.1	12	14.0	67	77.9	0.131
Medium nicotine dependence	5	13.2	8	21.1	25	65.8	
High nicotine dependence	4	30.8	2	15.4	7	53.8	

Although the majority of physicians and nurses believed that they had the necessary knowledge to counsel patients on how to quit smoking (85.7 % vs 92.6 % respectively, *p* = 0.052), those (doctors and nurses) who felt more confident about their knowledge regarding smoking cessation, reported counseling their patients to quit smoking more often compared to those who did not (92 % vs 60 %, *p* < 0.001).

## Discussion

The present study examined the smoking behavior among Cypriot physicians and nurses working in NGH, which is the largest tertiary hospital of Cyprus. Smoking prevalence among physicians and nurses was slightly higher (28.6 and 28.1 %) compared to that of the general population (26.5 %) [9]. Smoking prevalence among healthcare professionals in this study was lower in comparison to results from other neighboring countries such as Greece [7, 19] (39 % among physicians and 46 % among nurses, respectively) and Turkey [14, 20] (38 % among physicians and 45 % among nurses, respectively). An encouraging finding was that the majority of the participants were classified as having low nicotine dependence (61.3 %), and 71.8 % of current smokers expressed the wish to quit.

Several countries have achieved a considerable decline in tobacco consumption among healthcare professionals in the last decades. In Cyprus however, it is not known whether smoking prevalence among physicians and nurses has increased or declined, since this is the first survey of its kind. According to some of the earliest epidemiological studies in the USA, almost 40 % of physicians smoked in 1959, a finding which has shown remarkable decline, reaching less than10% by the mid-1990s [14]. Contemporary data reveal that only 2 % of American physicians smoke [14]. A similar downward trend was also seen in the UK, where smoking rates among British doctors fell from 62 to 18 % during the period 1951–1990 [21]. A survey performed in 2011 indicated that only 7 % of doctors were current smokers, which is a significantly lower rate than in the general population (24 %) [22]. On the contrary, 45 % of Chinese and 43 % of Japanese physicians respectively, were identified as current smokers [12]. Similar results were also reported for Kuwait (38 %), Estonia (24.9 % for males and 10.8 % for female doctors) and France (34 %) [14]. Smoking rates have also declined among nurses during the last decades, this decline however, was of a smaller magnitude compared to doctors whereas, in some countries the percentage of nurses who smoke is still very high [8]. Findings from Spanish and Italian studies reported high smoking prevalence among nurses (28 % [23] and 41 % [20, 24] respectively), while according to a survey in Britain, in 2011, nurses were more likely to be current smokers (8.7 %) than doctors (7 %) [22].

Results of the present study showed that gender, age, marital status and family history of smoking were significantly and independently associated with smoking status, whereas occupation (physician vs. nurse) was not found to be associated with smoking status. This is consistent with results of other studies that showed that male and unmarried physicians [6, 7, 11, 25] and nurses [26], with a family history of tobacco use, were more likely to become smokers. Both physicians and nurses reported that their main motive to quit smoking was health problems, which is consistent with results from other studies [6, 7, 11, 25]. Nearly all surveys that have studied the smoking habits of healthcare professionals, showed that a high percentage had made at least one attempt for smoking cessation [7, 11, 19, 27, 28]. In this study, about half of physicians (48.5 %) and nurses (51.9 %) who were smokers had attempted to quit smoking in the past. An interesting finding was that physicians reported a significantly higher success rate compared to nurses (62.5 % vs. 31.5 %). Nevertheless, only 6.5 % of the participants used smoking cessation programs in their attempt to quit smoking questioning thus the availability and usefulness of such programs in Cyprus.

Other studies have suggested that the majority of physicians and nurses agree that their smoking behavior possibly influences their attitude towards counseling patients to quit smoking [19, 28, 29]. A study regarding smoking prevalence of qualified nurses in Northern Ireland, found that nurses who continue to smoke were less willing to take on the role of a health promoter towards patients who smoke [30]. This finding is supported by the results of the present study showing that current smokers discuss less often with their patients about smoking cessation interventions, compared to former and never-smokers. In addition, nurses counseled their patients more often than physicians. This may be attributed to the fact that nurses spend more time with the patients and feel that counseling may be part of their responsibilities. A study which was performed in San Francisco in 1990, found that nurse practitioners discussed smoking with patients more often than did physicians (64 % vs. 50 %; *p* < 0.01), asked patients more often whether they were interested in quitting (49 % vs. 40 %; *p* < 0.01), distributed more smoking-cessation literature to patients (37 % vs. 25 %; *p* < 0.01) and made more follow-up appointments (36 % vs. 19 %; *p* < 0.01) [31]. In contrast, an Australian study reported that nurses are too busy to discuss with their patients about smoking cessation [26].

Studies in Italian [27] and Chinese [32] populations revealed that chest physicians and healthcare providers respectively, reported lack of formal medical teaching about smoking cessation strategies. Similarly, in Bosnia-Herzegovina more than half of the healthcare professionals reported that they were inadequately prepared to counsel patients [29]. However, in our study the majority of both physicians and nurses felt confident about their knowledge regarding smoking cessation interventions. This is encouraging, since data suggest that healthcare professionals who felt confident about their knowledge, counsel their patients to quit smoking more often. An encouraging finding of this study was the fact that current smokers were positive towards receiving specialized training for smoking cessation, during their career. However, there was a group of doctors who seem to ignore the harmful properties of smoking and admitted that they do not recommend smoking cessation in situations like lung problems (7.5 %), use of contraception pills (44.8 %), damage in mouth and lips (47.8 %), pregnancy (13 %), nervousness and anorexia (58.2 %), and insomnia (59.7 %). This could be attributed to possible knowledge gaps about the destructive effects of smoking or due to their own smoking behaviors. The latter finding is in line with other studies reporting that many healthcare professionals smoke in front of their patients [7, 25, 28] and agree with the statement that smoking is not as harmful as experts declare [11]. In our study, only a small group of participants reported smoking in front of their patients (5.9 % of physicians and 8 % of nurses respectively), indicating failure to fulfill their function as role models for their patients and society.

In the context of harmonization of Cypriot legislation with European directives on smoking, the provisions of the law “Protection of Health-Tobacco Control” were applied in 2010. The law specifically prohibits smoking in recreational areas and in enclosed public places. However, the main problem observed is the failure to practically apply the legislation in most areas, even in hospitals. Healthcare professionals who are smokers, still smoke even at the hospital setting. So far additional measures to ban smoking have not been taken among healthcare professionals, however the Ministry of Health will proceed with the preparation of Regulations in an effort to improve legislation and enhance the efforts for its implementation [33].

### Limitations of the study

One important limitation of the study is the cross-sectional design that only measures the social phenomenon at a specific moment in time and we were thus unable to measure the level of behavioral change concerning smoking habits. Nevertheless, having enough sample size, the aims of the study could be achieved with a cross-sectional design. In addition, smoking prevalence among physicians and nurses is likely to be underestimated, as data were self-reported. Physicians and nurses are perceived as most knowledgeable about the devastating effects of smoking; therefore, they may be prone to self-deception or understatement (misclassification bias). However, the anonymity of the questionnaire supports the possibility of the responses being true. Furthermore, the sample collection was limited to NGH and doctors and nurses from other hospitals in Cyprus did not participate in the study. It is worth noting that the NGH employs almost half of the physicians and nurses that work in the public healthcare sector of Cyprus, and the sample is likely to be representative; nevertheless, one should be cautious about generalizing the findings to all Cypriot physicians and nurses.

## Conclusions

According to the results of this study, smoking prevalence among healthcare professionals was 28.2 % in Cyprus, reflecting the prevalence of the general population. Factors associated with smoking status were age, gender, marital status and family history of smoking. Regarding the attitudes of healthcare professionals towards counseling patients to quit smoking, the study showed that their personal smoking behavior affected their intention to counsel patients to quit smoking. The results of this study support the need for further training of healthcare professionals towards smoking cessation strategies in order to improve their knowledge and consequently their efforts of providing counseling and support to their patients who wish to quit smoking.

### Ethics approval and consent to participate

According to the Cyprus National Bioethics Committee the study does not fall within the competence of the Cyprus National Bioethics Committee for bioethical review.

## Abbreviations

FTQ: Fagerstrom Nicotine Tolerance Questionnaire
NGH: Nicosia General Hospital
WHO: World Health Organization
